# Supplementary material for: Mediator subunit Med15 dictates the conserved “fuzzy” binding mechanism of yeast transcription activators Gal4 and Gcn4
Source: Nat Commun. 2021 Apr 13;12:2220. doi: 10.1038/s41467-021-22441-4 (PMC8044209; doi:10.1038/s41467-021-22441-4)
Supplement: Supplementary file 1 — Supplementary Information [file 41467_2021_22441_MOESM1_ESM.pdf]

a

Hyd. Region 1   Hyd. Region 2

828   840   855   870

```
Scerevisiae     TASKIDDGNNSKPLSPGWTDQTAYNAFGI-TTGMFNTTTMDDVYNYLFDDEDTPPNPKKE------------
Smikatae        TASKIDDDDNSKLLAPSWTDQTAYNAFGI-TAGMFNTTTMDDVYNYLFDDDDTPPNPINE------------
Skudriavzevii   TTSKPD-DDNSKSLSLNWTDQTAYNAFGN-TTGMFNTTTMDDVYNYLFDDDDTPPNPSLNEL----------
Seubayanus      ATSNFD-DDNSKSMFPNWTDQTAYNALGI-TTGMFNTTTMDDVYNYLFDDDETPPNLNSE------------
Sbayanus        ATNNVD-DDNSKSLFPNWTDQTAYNALGI-TTGMFNTTTMDDVYNYLFDDDETPPNLNSE------------
Zrouxii         PNSAIKTGHPLDNEGPFWTDQAAYNAFGL-TSSLFNTTTMDDVYNFLFDEEDHTPPKSKSWQDSDRH-----
Ndairenensis    -NNSNMNQDLTKVPFHGWNDQTAYNAFGM-TSGMFNTTTMDDVYNYLFDNDDISQDVTASTNVSKSNNN---
Scastellii      QQQQMATTDPNKTLFPSWNDQTAYNAFGM-TSGMFNTTTMDDVYSYLFDTDELTPPNVNLNS----------
Tblattae        QNTDNQNTEPSKAILPIWADQTAYSTFGI-APTMFNTTTMDDVYNYLFDDQETPPNANNAANLTSTYTSPKI
Lthermotol      -SSSAQPLKQDATVFPNWNDQSVQTLFNPGTSNMFNTTTMDDIFNYVFNDNELAPSISNSSNQS--------
Klactis         ------TTQMRPPTTSGWPDTNN--FLNPSTQQLFNTTTMDDVYNYIFDNDE--------------------
Knaganishii     LMNNPLPGNPGINSNLNWNDNSAFSTVGF-TNGLFNTTTMDDVYNYLFDDNTTVQNMPSSNIVSDVTASERT
Kafricana       LT--RSPNQQFNGGNINWNDNTALNALGL-TGGLFNTTTMDDVYNYLFDDNVSNSTNSS-------------
Conservation            .        * *      .   :  :********::.::*: :
```

b

```
Gal4 AD   828-DDGNNSKPLSPGWTDQTAYNAFGITTGMFNTTTMDDVYNYLFDDEDTPPNPKKE-881
Gcn4 cAD                      101-SSTDSTPMFEYENLEDNSKEWTSLFDNDIPVTTDD-134
```

Supplementary Figure 1. Related to Figures 1 and 2. **Conservation of the yeast Gal4 AD. a** Clustal omega alignment of yeast Gal4 AD sequences. Residues that, when mutated to either Ala or Pro, inhibit Gal80-mediated repression are boxed [1,2]. Hydrophobic regions I and II are indicated with brackets. **b** Gal4 AD sequence manually aligned with Gcn4 cAD WxxLF. Color code is as follows: Red, hydrophobics; Green, hydrophilics; Magenta, basics; Blue, acidics.

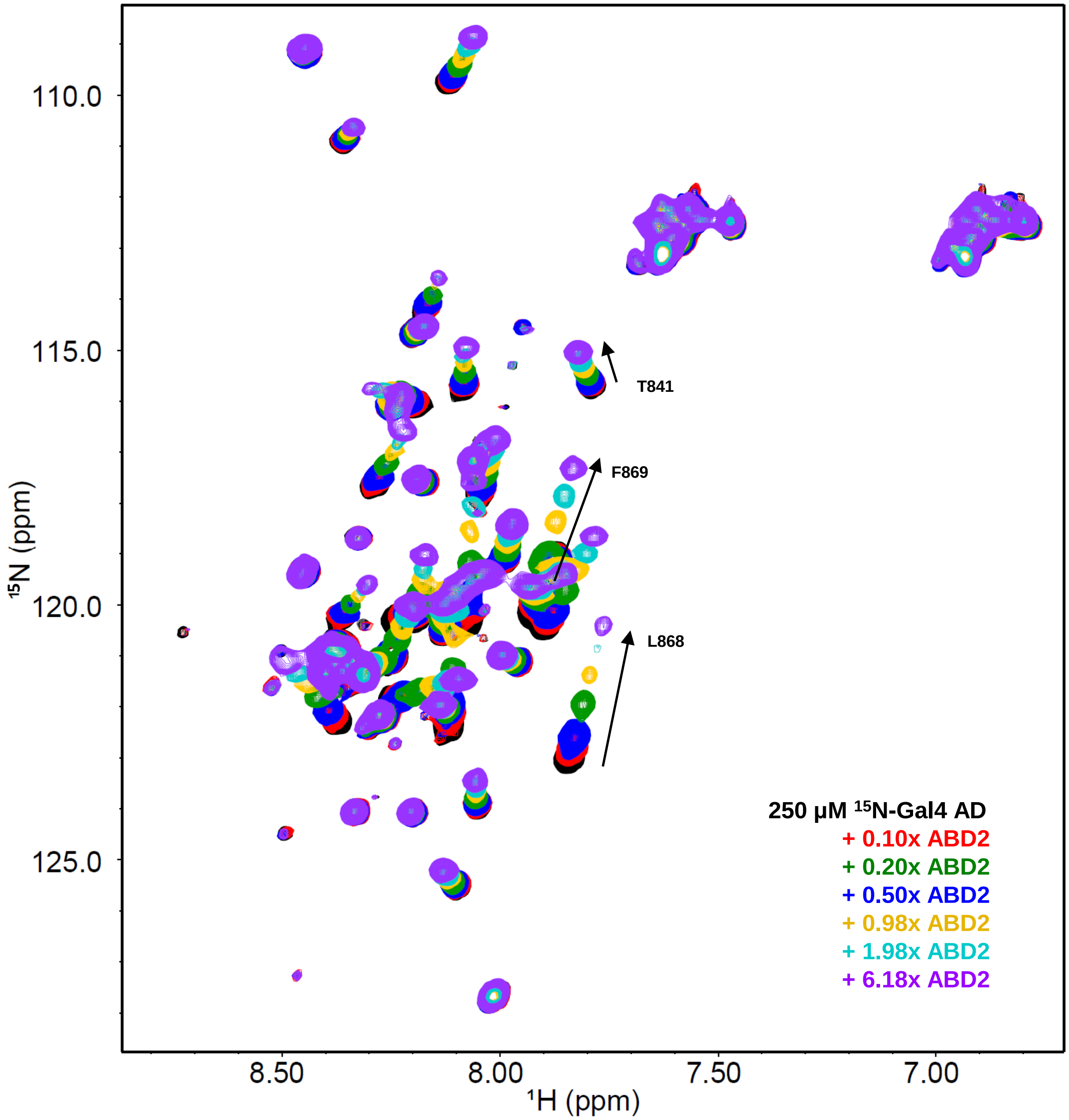

Supplementary Figure 2. Related to Figure 3. **Titration of Gal4 AD and ABD2.** (^{1}H,^{15}N)-HSQC titration spectra of ^{15}N-Gal4 AD with ABD2. Fraction saturation based on the K_d are 0 (black), 0.08 (red), 0.17 (green), 0.41 (blue), 0.68 (yellow), 0.89 (cyan), and 0.97 (purple).

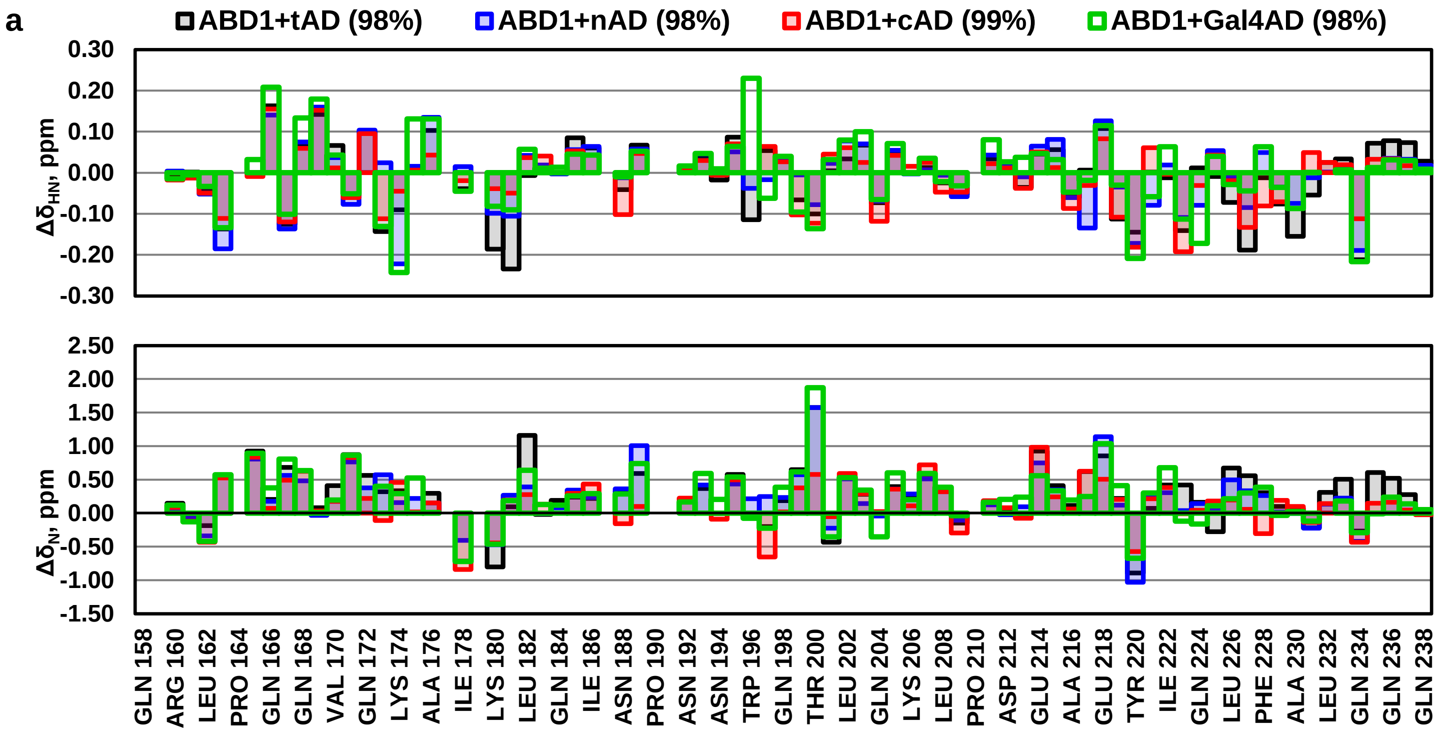

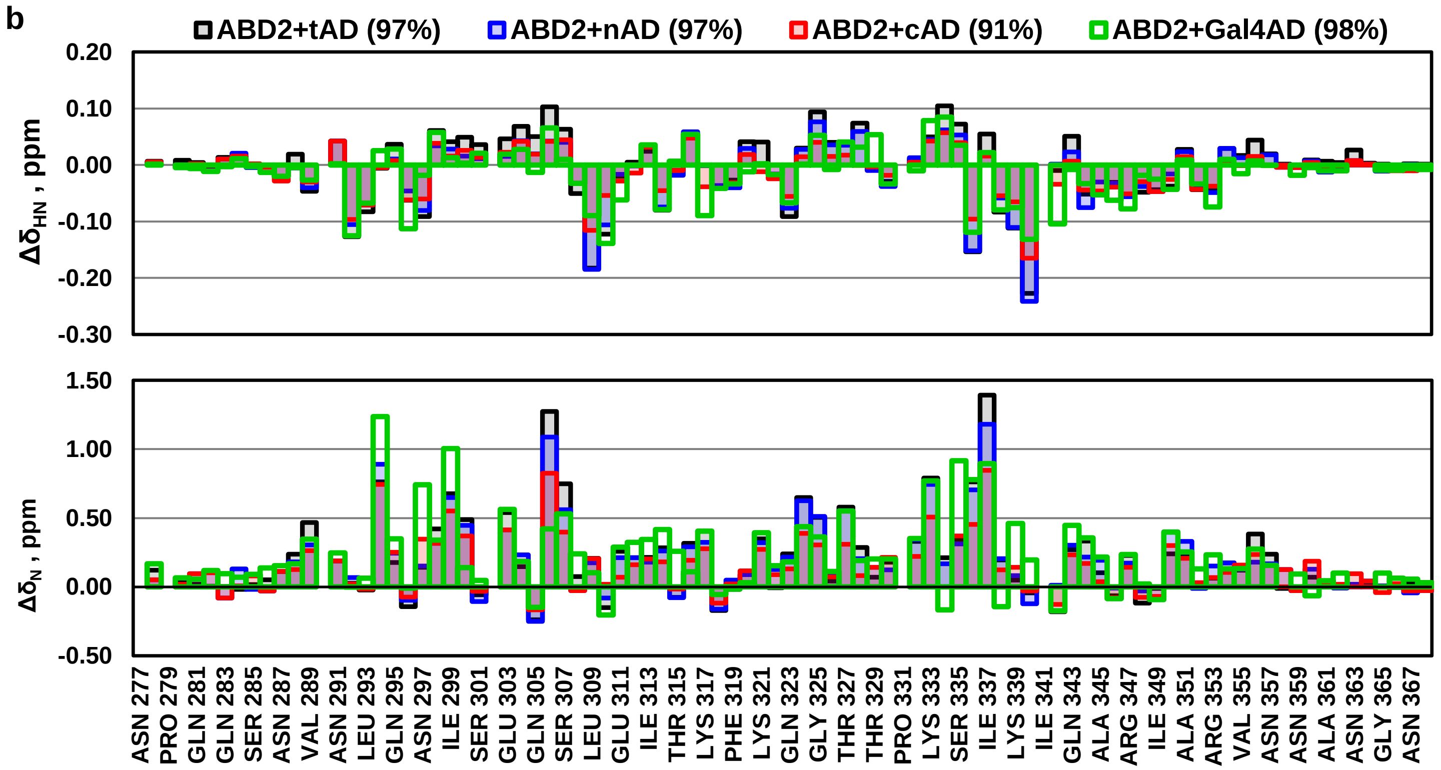

Supplementary Figure 3. Related to Figure 4. **CSPs of ABD1 and ABD2 with ADs. a** ABD1 and **b** ABD2 show widespread backbone amide chemical shift perturbations (CSPs) with each AD. Gcn4 nAD (residues 1-100), cAD (101-134), and tAD (1-134) show strikingly similar CSPs as for Gal4 AD. Percent saturation for each titration is given in parentheses in the legend entry. Gcn4 data is from Tuttle et al., 2018 [3].

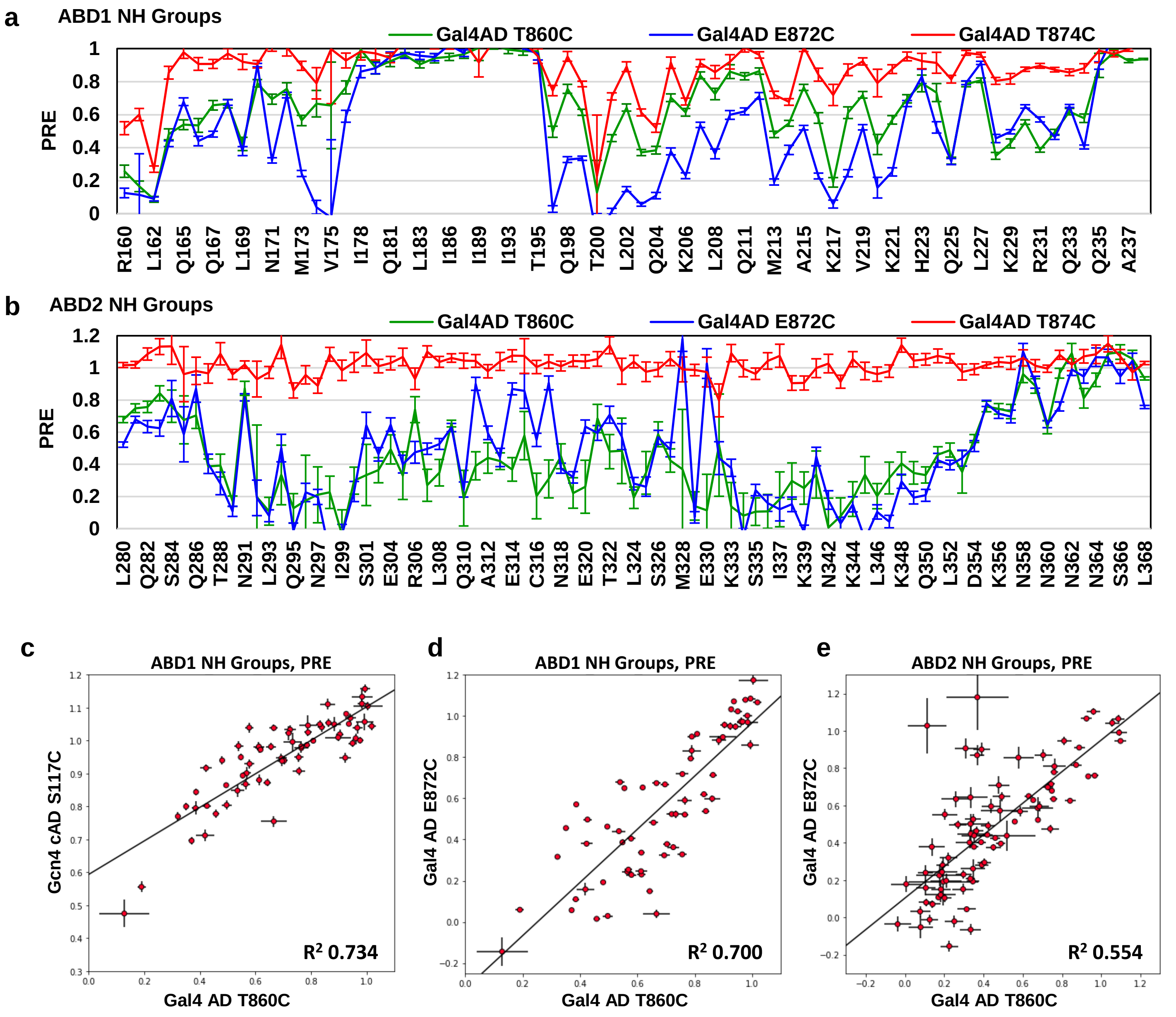

Supplementary Figure 4. Related to Figure 5. **Paramagnetic Relaxation Effects (PRE) on ABD1 and ABD2 when bound to spin-labeled Gal4 AD.** Tempo spin-label was attached at Gal4 AD position T60C, E872C, or T874C. PRE of each spin-labeled AD is shown for groups for **a** ABD1 and **b** ABD2. Error bars represent standard error based spectral noise intensities. **c** PREs for ABD1+ Gal4 AD are significantly correlated with those of ABD1 + Gcn4 cAD S117C-TEMPO. **d-e** PRES for ABD1 and ABD2 correlate well for spin-labels at either Gal4 AD T860C or E872C. The true relationship between PRE data is not expected to be linear, but a linear fit demonstrates a strong correlation of the data. Gcn4 data is from Tuttle et al., 2018 [3].

Supplementary Table 1. Primers Used

plasmid	mutation	mutagenesis oligo name	sequence
pSH1687	840-860	Gal4_d861-881t	cagggatgtttaataccactacaGGTTCTGGTTCCGGTTCTGACAATGACATTC
pSH1687	840-860	Gal4_d861-881b	GAATGTCATTGTCAGAACCGGAACCAGAACCtgtagtggtattaaacatccctg
pSH1904	828-871	Gal4_d872-881t	gatgtatataactatctattcgatgatGGTTCTGGTTCCGGTTCTGACAATGACATTC
pSH1904	828-871	Gal4_d872-881b	GAATGTCATTGTCAGAACCGGAACCAGAACCatcatcgaatagatagttatatacatc
pSH1511	Y865A	Gal4 865At	ccactacaatggatgatgtaGCTaactatctattcgatgatgaag
pSH1511	Y865A	Gal4 865Ab	cttcatcatcgaatagatagttAGCtacatcatccattgtagtgg
pSH1512	Y867A	Gal4 867At	caatggatgatgtatataacGCTctattcgatgatgaagataccc
pSH1512	Y867A	Gal4 867Ab	gggtatcttcatcatcgaatagAGCgttatatacatcatccattg
pSH1515	M2	Gal-Gcn_M2t	taaaatgtggacggaccaaactgcgGCTaacgcgGCTggaatcactacagggatgtttaatacc
pSH1515	M2	Gal-Gcn_M2b	ggtattaaacatccctgtagtgattccAGCcgcgttAGCcgcagtttggtccgtccacattta
pSH1516	M3	Gal-Gcn_M3t	aacgcgtttggaatcactacagggGCTGCTaataccactacaatggatgatg
pSH1516	M3	Gal-Gcn_M3b	catcatccattgtagtggtattAGCAGCccctgtagtgattccaaacgcgtt
pSH1513	L868A	Gal4 868At	caatggatgatgtatataactatGCTttcgatgatgaagataccccacc
pSH1513	L868A	Gal4 868Ab	ggtggggtatcttcatcatcgaaAGCatagttatatacatcatccattg
pSH1514	F869A	Gal4 869At	caatggatgatgtatataactatctaGCTgatgatgaagataccccacc
pSH1514	F869A	Gal4 869Ab	ggtggggtatcttcatcatcAGCtagatagttatatacatcatccattg
pSH1519	847-881	Gal-Gcn d1t	GAAAATAAATTAAATACAAATAAAATGaacgcgtttggaatcactacaggg
pSH1519	847-881	Gal-Gcn d1b	ccctgtagtgattccaaacgcgttCATTTTATTTGTATTTAATTTATTTTC
pSH1520	852-881	Gal-Gcn d2t	GAAAATAAATTAAATACAAATAAAATGactacagggatgtttaataccactac
pSH1520	852-881	Gal-Gcn d2b	gtagtggtattaaacatccctgtagtCATTTTATTTGTATTTAATTTATTTTC
pSH1521	857-881	Gal-Gcn d3t	GAAAATAAATTAAATACAAATAAAATGaataccactacaatggatgatg
pSH1521	857-881	Gal-Gcn d3b	catcatccattgtagtggtattCATTTTATTTGTATTTAATTTATTTTC
pSH1518	M5	Gal-Gcn_M2+3t	taaaatgtggacggaccaaactgcgGCTaacgcgGCTggaatcactacagggGCTGCTaataccactacaatggatgatg
pSH1518	M5	Gal-Gcn_M2+3b	catcatccattgtagtggtattAGCAGCccctgtagtgattccAGCcgcgttAGCcgcagtttggtccgtccacattta
pSH1985	M4b	Gal4 840_46_49_At	gaaaataaattaaatacaaataaaatgGCTacggaccaaactgcgGCTaacgcgGCTggaatcactacagggatgtttaatacc
pSH1985	M4b	Gal4 840_46_49_Ab	ggtattaaacatccctgtagtgattccAGCcgcgttAGCcgcagtttggtccgtAGCcattttatttgtatttaatttattttc
pSH1531	YLF->AAA	Gal4 YLFt	caatggatgatgtatataacGCTGCTGCTgatgatgaagataccccac
pSH1531	YLF->AAA	Gal4 YLFb	gtggggtatcttcatcatcAGCAGCAGCgttatatacatcatccattg
pSH1973	871C	Gal4_871Ct	ggatgatgtatataactatctattcgatTGCgaagataccccaccaaacccaaaaaaagagTAAAATGGAGCTCCGTCG
pSH1973	871C	Gal4_871Cb	CGACGGAGCTCCATTTTActctttttttgggtttggtggggtatcttcGCAatcgaatagatagttatatacatcatcc
pSH1974	872C	Gal4_872Ct	ggatgatgtatataactatctattcgatgatTGCgataccccaccaaacccaaaaaaagagTAAAATGGAGCTCCGTCG
pSH1974	872C	Gal4_872Cb	CGACGGAGCTCCATTTTActctttttttgggtttggtggggtatcGCAatcatcgaatagatagttatatacatcatcc
**RTqPCR primers**	ACT1	ACT1-FP1	TGG ATT CCG GTG ATG GTG TT
		ACT1-RP1	TCA AAA TGG CGT GAG GTA GAG A
	ARG3	F-RT-ARG3	TCGCATGTCTGAAATTCGGTAT
		R-RT-ARG3	CATCGACAATATCGGAATCCATT
	HIS4	HIS4-FP1	GCA CTG CCA TTT TAC CAA GTA CTG
		HIS4-RP1	CTT GGT GGA GAT GCA AAC ACA

Supplementary References

1. Salmeron, J.M., Jr., Leuther, K.K. & Johnston, S.A. GAL4 mutations that separate the transcriptional activation and GAL80-interactive functions of the yeast GAL4 protein. *Genetics* **125**, 21-7 (1990).
2. Ansari, A.Z., Reece, R.J. & Ptashne, M. A transcriptional activating region with two contrasting modes of protein interaction. *Proc Natl Acad Sci U S A* **95**, 13543-8 (1998).
3. Tuttle, L.M. et al. Gcn4-Mediator Specificity Is Mediated by a Large and Dynamic Fuzzy Protein-Protein Complex. *Cell Rep* **22**, 3251-3264 (2018).
