# Supplementary material for: Mediator subunit Med15 dictates the conserved “fuzzy” binding mechanism of yeast transcription activators Gal4 and Gcn4
Source: Nat Commun. 2021 Apr 13;12:2220. doi: 10.1038/s41467-021-22441-4 (PMC8044209; doi:10.1038/s41467-021-22441-4)
Supplement: Supplementary file 3 — Reporting summary [file 41467_2021_22441_MOESM3_ESM.pdf]

nature research

Corresponding author(s): Rachel E Klevit

Last updated by author(s): Mar 1, 2021

Reporting Summary

Nature Research wishes to improve the reproducibility of the work that we publish. This form provides structure for consistency and transparency in reporting. For further information on Nature Research policies, see our Editorial Policies and the Editorial Policy Checklist.

Statistics

For all statistical analyses, confirm that the following items are present in the figure legend, table legend, main text, or Methods section.

n/a	Confirmed	
x		The exact sample size (*n*) for each experimental group/condition, given as a discrete number and unit of measurement
	x	A statement on whether measurements were taken from distinct samples or whether the same sample was measured repeatedly
x		The statistical test(s) used AND whether they are one- or two-sided<br>*Only common tests should be described solely by name; describe more complex techniques in the Methods section.*
x		A description of all covariates tested
x		A description of any assumptions or corrections, such as tests of normality and adjustment for multiple comparisons
	x	A full description of the statistical parameters including central tendency (e.g. means) or other basic estimates (e.g. regression coefficient) AND variation (e.g. standard deviation) or associated estimates of uncertainty (e.g. confidence intervals)
x		For null hypothesis testing, the test statistic (e.g. *F*, *t*, *r*) with confidence intervals, effect sizes, degrees of freedom and *P* value noted<br>*Give P values as exact values whenever suitable.*
x		For Bayesian analysis, information on the choice of priors and Markov chain Monte Carlo settings
x		For hierarchical and complex designs, identification of the appropriate level for tests and full reporting of outcomes
x		Estimates of effect sizes (e.g. Cohen's *d*, Pearson's *r*), indicating how they were calculated

Our web collection on statistics for biologists contains articles on many of the points above.

Software and code

Policy information about availability of computer code

Data collection	Bruker Topspin 3.2 for all NMR data, Xplor-NIH version 3.0-rc3 for structure simulations
Data analysis	NMRPipe 8.9 (Delaglio et al., 1995); NMRviewJ 9.0.2 (Johnson, 2004); Pymol 1.7 (Schrodinger, 2015); Microcal Origin 7.0 Origin Lab Corporation; Prism 7 Graphpad Software, Inc; Jupyter Notebook 6.0.3 (Kluyver et al., 2016)

For manuscripts utilizing custom algorithms or software that are central to the research but not yet described in published literature, software must be made available to editors and reviewers. We strongly encourage code deposition in a community repository (e.g. GitHub). See the Nature Research guidelines for submitting code & software for further information.

Data

Policy information about availability of data

All manuscripts must include a data availability statement. This statement should provide the following information, where applicable:

- Accession codes, unique identifiers, or web links for publicly available datasets
- A list of figures that have associated raw data
- A description of any restrictions on data availability

NMR chemical shifts have been deposited to the BioMagResBank: Gal4 AD 828-881 (accession number 50086). Figures 2, 3, 5 and Supplementary Figures 3 and 4 have associated raw data in the Source Data.xlsx file.

Field-specific reporting

Please select the one below that is the best fit for your research. If you are not sure, read the appropriate sections before making your selection.

☒ Life sciences ☐ Behavioural & social sciences ☐ Ecological, evolutionary & environmental sciences

For a reference copy of the document with all sections, see nature.com/documents/nr-reporting-summary-flat.pdf

Life sciences study design

All studies must disclose on these points even when the disclosure is negative.

Sample size	RTqPCR assays were done in biological duplicate and technical triplicate. With this approach, our SEM for values of HIS4 transcription are ≤ 6.5% and ≤25.5% at ARG3.
Data exclusions	no exclusions
Replication	Transcription activity assays were performed in duplicate; all attempts at replication were successful.
Randomization	Gal4 activation domain derivatives tested were not randomized. Derivatives were designed to test the function of residues in the two hydrophobic clusters of the AD. Both alanine substitutions and deletions were used to test the biological function of these hydrophobic regions in transcription activation.
Blinding	No blinding: Based on our experimental approach that includes testing all samples in bulk without regard for identity of the samples, we feel that observer bias is not a concern.

Reporting for specific materials, systems and methods

We require information from authors about some types of materials, experimental systems and methods used in many studies. Here, indicate whether each material, system or method listed is relevant to your study. If you are not sure if a list item applies to your research, read the appropriate section before selecting a response.

Materials & experimental systems

n/a	Involved in the study	
☐	☒	Antibodies
☐	☒	Eukaryotic cell lines
☒	☐	Palaeontology and archaeology
☒	☐	Animals and other organisms
☒	☐	Human research participants
☒	☐	Clinical data
☒	☐	Dual use research of concern

Methods

n/a	Involved in the study	
☒	☐	ChIP-seq
☒	☐	Flow cytometry
☒	☐	MRI-based neuroimaging

Antibodies

Antibodies used	None; no antibodies used in data reported in final form of manuscript
Validation	*Describe the validation of each primary antibody for the species and application, noting any validation statements on the manufacturer's website, relevant citations, antibody profiles in online databases, or data provided in the manuscript.*

Eukaryotic cell lines

Policy information about cell lines

Cell line source(s)	S cerevisiae; SHY944
Authentication	Authenticated by PCR and genetic analysis for Gcn4, Gal80 deletions and by genetic assays for other auxotrophic mutant phenotypes.
Mycoplasma contamination	not relevant for yeast
Commonly misidentified lines (See ICLAC register)	none
